# Microfluidic Chip with Fiber-Tip Sensors for Synchronously Monitoring Concentration and Temperature of Glucose Solutions

**DOI:** 10.3390/s23052478

**Published:** 2023-02-23

**Authors:** Jian Qu, Yi Liu, Yan Li, Jinjian Li, Songhe Meng

**Affiliations:** 1 Center for Composite Materials, Harbin Institute of Technology, Harbin 150001, China; 2 School of Physics, Harbin Institute of Technology, Harbin 150001, China

**Keywords:** microfluidic chips, fiber-tip sensor, dual parameter sensor

## Abstract

Monitoring the properties of fluids in microfluidic chips often requires complex open-space optics technology and expensive equipment. In this work, we introduce dual-parameter optical sensors with fiber tips into the microfluidic chip. Multiple sensors were distributed in each channel of the chip, which enabled the real-time monitoring of the concentration and temperature of the microfluidics. The temperature sensitivity and glucose concentration sensitivity could reach 314 pm/°C and −0.678 dB/(g/L), respectively. The hemispherical probe hardly affected the microfluidic flow field. The integrated technology combined the optical fiber sensor with the microfluidic chip and was low cost with high performance. Therefore, we believe that the proposed microfluidic chip integrated with the optical sensor is beneficial for drug discovery, pathological research and material science investigation. The integrated technology has great application potential for micro total analysis systems (μ-TAS).

## 1. Introduction

Micro total analysis systems (μ-TAS) equipped with various functional devices have been rapidly developed in recent years. Samples can be precisely controlled in microchannels, reaction chambers and other functional components used in research fields such as biomedicine, chemistry, pharmacy, environmental science and bionics [1,2,3,4]. Microfluidic switches [5], microvalves [6], micronanocounters [7], flow meters [8], nanorobots [9] and micronanostructures [10] have been studied and used in μ-TAS. Organs on chips have also been investigated [11,12,13,14]. Combined with the methods for simulating and monitoring various living cells, tissues and organs on chips, they reflect the main structural and functional features of living tissues and organs. Optofluidic devices are well suited for the overall analysis in microspace because they can integrate sample preparation and delivery with the analysis mechanism. Fiber sensors have the distinct advantages of corrosion resistance, small size, antielectromagnetic interference and biocompatibility based mainly on Mach–Zehnder interferometers (MZIs) [15], Fabry–Pérot interferometers (FPIs) [16], surface plasmon resonances (SPRs) [17], localized surface plasmon resonances (LSPRs) [18] and fiber gratings [19,20]. In 2019, Zhong et al. presented a D-type plastic fiber sensor with three evanescent wave probes for the accurate online measurement of the progression and phenol degradation of a microalgal biofilm. The *Chlorella vulgaris* biofilm was deposited on the plastic fiber with a large size [21]. Guan et al. designed an optofluidic MZI for the online monitoring of photocatalytic reactions in a single channel [22]. The temperature compensation structure should be designed to obtain the accurate detection value. An FP Resonator is also used in marine phosphate detection with intensity modulation embedded on both sides of the channel. Additionally, the microfluidics to be tested are even injected into the optical hollow fiber. Moreover, fiber Bragg gratings (FBGs) and SPR fiber sensors are used in microfluidic chips as monitoring detectors [23]. However, these point detection sensors can only achieve a low detection rate and are prone to errors when integrating devices and microfluidic chips.

In recent years, more and more people have been suffering from hyperglycemia. Hyperglycemia means that the glucose level in the blood is higher than the normal value and the blood glucose is higher than 7.7 mmol/L 2 h after meals [24,25]. Several glucose sensors based on a special interference structure, SPR and LSPR, have been proposed [26,27,28,29]. However, there is still a cross between the concentration of the glucose solution and temperature measurement, which affects the accuracy of the measurement results. In addition, most glucose sensors are point monitoring systems, which makes it difficult to monitor the overall concentration distribution. Therefore, an effective glucose concentration monitoring system is needed.

In this work, a microfluidic chip equipped with high-sensitivity dual-parameter fiber-tip sensors is proposed. The effects of different probe shapes on the fluid field in microchannels were investigated. Hemispherical probes were distributed in each channel of the chip, allowing for the synchronous real-time monitoring of the concentration and temperature of the solutions. The temperature sensitivity and glucose concentration sensitivity could reach 314 pm/°C and −0.678 dB/(g/L), respectively. The hemispherical cap sensor probe (HCSP) hardly affected the microfluidic flow field, and the flow field recovered quickly. The chip did not react with the microfluidic due to its chemical inertness, which is a great advantage for the microfluidic chip. Therefore, we believe that microfluidic chip-embedding functional devices have great application potential in biomedical and pharmaceutical engineering. The functional device embedding technology will provide a new method for functional device integration research.

## 2. Principles and Fabrication

### 2.1. Principles

**Figure 1:**
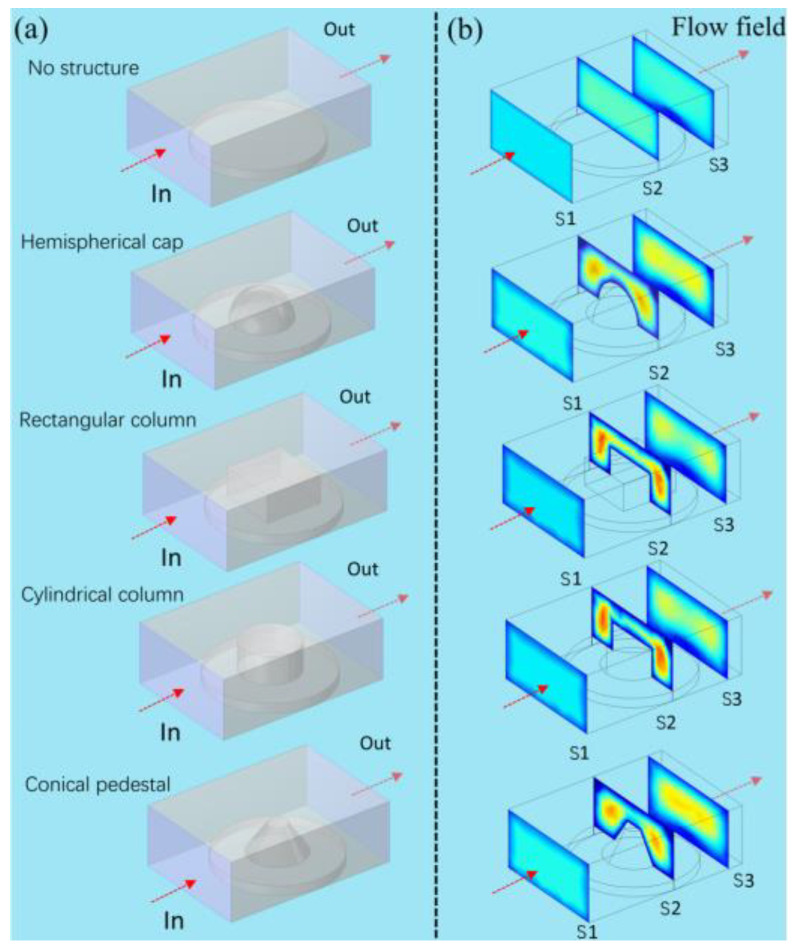
(**a**) Analysis diagram of four structures: hemispherical cap, rectangular column, cylindrical column and conical pedestal. (**b**) The flow field distribution.

The microfluidic flow field in the channel is affected when the fiber optic sensor with a different shape is integrated into the microfluidic chip. Finite element analysis (FEA) was used to analyze the effects of the shape of the probe on the flow velocity field. The hemispherical cap, rectangular column, cylindrical column and conical base were designed and simulated at certain flow rates as shown in Figure 1a. The fluid flowed over the probe in the microchannel and the flow field was affected by the probe. Three sections of the flow velocity field were chosen at different positions in the channel, as shown in Figure 1b. S1 was the section that the fluid passed before the probe, S2 was the section where the fluid flowed over the probe and S3 was the section that the fluid passed after the probe. The flow velocity field was divided into two parts as the fluid flowed over the probe. The flow velocity field recovered faster when the fluid flowed over the hemispherical cap probe and the conical base probe than the other probes. Moreover, the hemispherical cap structure could be fabricated without using the femtosecond laser two-photon polymerization technique [30], while the prepared triumph arc structure was not suitable for microfluidic chips. The HCSP could be fabricated by using the low-cost fiber end-face dispensing method.

**Figure 2:**
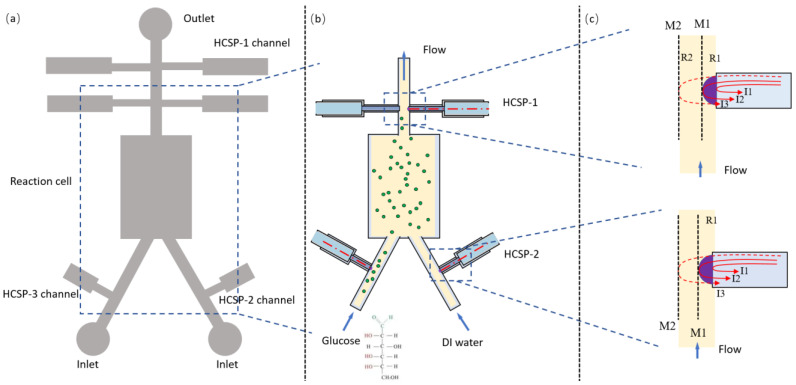
(**a**) Schematic diagram of the microfluidic chip design. (**b**) Optical fibers inserted in the fiber channels at the cross section. (**c**) Principle of light field interference of HCSP. M1: mirror 1, M2: mirror 2.

A microfluidic chip was designed and fabricated as shown in Figure 2a. In the chip, two channels with a width of 600 μm were provided as the inflow channels for the glucose solutions and deionized water (DI water), which were connected to the central reaction cell to ensure uniform mixing. The mixed solution drained through a main channel with a width of 600 μm. Several fiber grooves were distributed in each channel. The tip of the HCSP was simply inserted into the channel to monitor the temperature and concentration characteristics of the liquid, as shown in Figure 2b. Moreover, the stepped sensor embedding grooves were designed to fix the bare fiber and cladding with a width of 200 μm and 500 μm, respectively, in the channel. The gaps between the fiber and the grooves were sealed with 535 thermosetting adhesives, which were insensitive to temperature after curing. The HCSPs were embedded in the channels using the high-precision five-dimensional displacement platform. The thermosetting adhesive 535 did not flow into the microfluidic channel, as shown in Figure 2c. The ultrafine tapered fibers were immersed in the 535 adhesives to fill the unfilled gaps. The HCSP was made of SU-8 based on our previous work [30]. The end surface of the tip structure, the fiber and the inner wall of the channel formed a three-beam interference, as shown in Figure 2c. Due to the relatively large size of the channel, the intensity of light reflected from the channel (M1) was weak enough and was not within the interference region, which could be ignored. The incident light was reflected from both the fiber end surface and the surface of the cap structure (M2), and the two reflected beams interfered and superimposed. The light transmitted from the first surface reached the second reflective surface after traveling a distance L (μm) in the polymer cap. Then, the light field was reflected and transmitted at the second surface. Therefore, the intensity of the reflected interference light of the hemispherical SU-8 film structure can be expressed as follows:

(1) $$\begin{array}{l} I_{FP}(\lambda )=R_{1}+{\left(1-R_{1}\right)}^{2}{\left(1-\alpha \right)}^{2}\\ \;\;\;\;-2\sqrt{R_{1}R_{2}}(1-R_{1})(1-\alpha )\cos(4\pi Ln_{2}/\lambda ) \end{array}$$

*R*_1_ and *R*_2_: the reflection indices of the end face of the fiber and the HCSP;

*α*: the transmission loss of light within a polymer film;

*n*_1_, *n*_2_ and *n*_3_: the refractive indices (RI) of the core of the fiber, SU-8 film and microfluidics.

The central wavelength of the FPI interference dip and free spectral range (FSR) (nm) can be expressed as:

(2) $$\lambda_{m}=\frac{4n_{2}L}{2m_{th}+1}$$

(3) $$FSR=\lambda_{m+1}-\lambda_{m}=\frac{\lambda_{m}\lambda_{m+1}}{2h\sqrt{n_{2}^{2}-n_{1}^{2}}}$$

*m* is the interference order of the interference dip.

It can be seen that *λ_m_* would be influenced by *n*_2_ and *L* (μm), which is the shifting of the interference dips. *N*_2_ and *L* will change with the temperature due to thermal expansion and the thermo-optic effect of the SU-8 photoresist. Thus, the relationship between the interference dip and the temperature is expressed as:

(4) $$\Delta \lambda_{m}=\frac{4n_{2}}{2m+1}\left(\frac{n_{2}dL}{dT}\cdot \Delta T+\frac{Ldn_{2}}{dT}\cdot \Delta T\right)$$

When the ambient temperature varies between 25–55 °C, the coefficient of thermal expansion (CTE) and thermo-optic coefficient (TOC) of the SU-8 film are 52 ppm/°C and −1.87 × 10^−4^/°C, respectively. According to Equation (4), the temperature sensitivity of the hemispherical structure is about 368 pm/°C. In addition, according to the Fresnel formula:

(5) $$r_{2}=\frac{n_{2}\cos\theta -n_{3}\cos\theta \prime }{n_{2}\cos\theta +n_{3}\cos\theta \prime }$$

The minimum value of the light intensity of the reflection spectrum of the HCSP can be expressed as:

(6) $$\begin{array}{l} I_{FP-dips}(\lambda )=R_{1}+\frac{{\left(1-R_{1}\right)}^{2}{\left(1-\alpha \right)}^{2}{\left(n_{2}-n_{3}\right)}^{2}}{{\left(n_{2}+n_{3}\right)}^{2}}\\ \;\;\;\;\;-\frac{2\sqrt{R_{1}}(1-R_{1})(1-\alpha )(n_{2}-n_{3})}{\left(n_{2}+n_{3}\right)} \end{array}$$

The TOC of the optical fiber material and the SU-8 photoresist are weak enough that the intensity of the interference dips mainly depends on *n*_3_. Therefore, the RI of the microfluidics can be monitored by demodulating the intensity change in the interference dips. As mentioned earlier, the average wavelength of the interference dips is mainly affected by temperature, and the intensity mainly depends on the concentration of the solution. The wavelength and intensity of the interference dips can be demodulated. Thus, temperature and concentration can be monitored simultaneously.

### 2.2. Fabrication

The fabrication process of the probe was described in detail in our previous works [30,31]. Here, the fabrication process was optimized. The cap structure at the end surface of the fiber was cured on the UV laser coupling stage, and the stronger power made the single photon polymerization more thorough and solid. In addition, a high-precision two-dimensional displacement platform was used to prepare the cap structure. The fiber clamp was fixed on the two-dimensional platform, and two pieces of apartment-cut optical fibers were clamped, one of which was dipped in an SU-8 adhesive. The end faces were separated by moving the sliding platform after contacting, and the SU-8 photoresist was applied to the end of the fiber. The fiber with the SU-8 photoresist was heated at 85 °C for 45 min in a tube furnace to evaporate the solvent. Then, the structure was polymerized by a UV laser coupling step, forming some active groups. The polymerized structure was heated again to crosslink and cure the epoxy group, which was catalyzed by these active groups.

**Figure 3:**
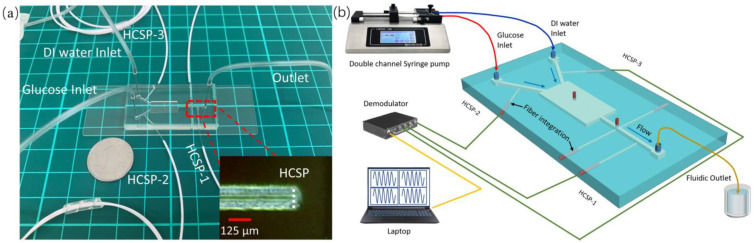
(**a**) View of the microchips connected to liquid pipes and HCSP, the insert is view of HCSP. (**b**) Experimental diagram of HCSP integrated microchips and demodulation system.

The fluid inlet and outlet were created by inserting steel capillaries into punched holes in the PDMS device. The sensor probe was prepared precisely and the size of the cladding and coating was consistent with the fiber channels. The 535 glue was injected into gaps between the fiber and grooves through the high-precision displacement platform. The multi HCSPs were inserted at the grooves as shown in Figure 3a. A high-concentration glucose solution and DI water were injected into the microfluidic channel through a dual-channel syringe pump (LSP01-2AY). The glucose concentration was controlled by controlling the flow rate and the injection time as shown in Figure 3b. Multiple fiber-optic sensing probes were connected to high-speed fiber grating demodulators with a coherent light sources to monitor the temperature and concentration of glucose in real time.

## 3. Results and Discussion

**Figure 4:**
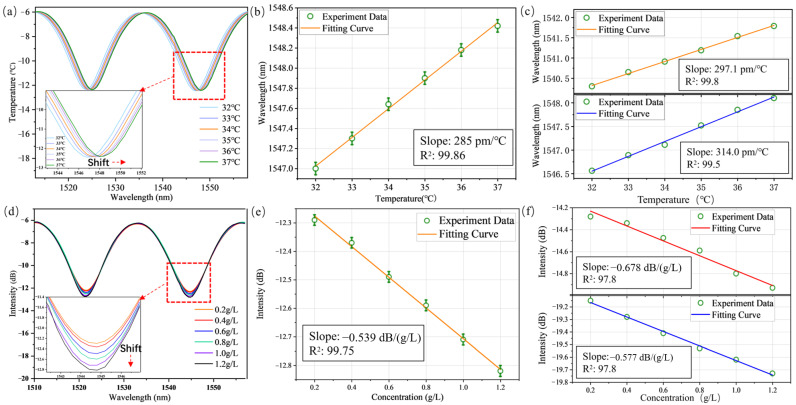
(**a**) Dips of FP interference reflection spectrum shifting with the temperature change. (**b**) The relationship between the dips shifts and the temperature variation in HCSP-1. (**c**) The temperature response of HCSP-2 and HCSP-3. (**d**) Dips of FP interference reflection spectrum shifting with the concentration of glucose solutions change. (**e**) The relationship between the intensity change in the interference dip and the concentration variation in HCSP-1. (**f**) The concentration response of HCSP-2 and HCSP-3.

The microchip was placed at a room temperature of 30 °C. DI water at a temperature of 32 °C to 37 °C in increments of 1 °C was injected into each channel. The interference slope of HCSP-1 gradually shifted toward the long wavelengths with increasing temperature, as shown in Figure 4a. To show the relationship between the wavelength shift and temperature in more detail, the interference dip at 1547 nm was selected in each structural spectrum and its central wavelength was recorded at different temperatures. The average central wavelength of the interference dips at each temperature was linearly fitted, and the temperature sensitivity of the hemispherical SU-8 film structure reached 285 pm/°C in the temperature range from 32 °C to 37 °C, which was consistent with Equation (4) as shown in Figure 4b. It can also be seen that the temperature sensitivity was mainly affected by the CTE of SU-8. The structure had good linearity in this temperature range with R^2^ of 99.86%, indicating that the probe had excellent stability. Compared with the conventional fused silica temperature sensor [19,20], the temperature sensitivity of the HCSP was much better due to the higher CTE of SU-8. The other two probes were also tested. The temperature sensitivities of HCSP-2 and HCSP-3 reached 297.1 pm/°C and 314 pm/°C, respectively, and the fitting curves had a high R^2^ of 99.8% and 99.5%, as shown in Figure 4c. The concentration response was also tested. As the concentration of the glucose solution increased, the loss intensity in the spectra increased. The interference dip at 1544.7 nm was selected to show the relationship between the loss intensity and concentration (see Figure 4e). The relationship curve was fitted linearly, and the R^2^ of the fitting curve reached 99.75%. The concentration sensitivity of the probe was −0.539 dB/(g/L) in the range of 0.2 g/L to 1.2 g/L.

The other two probes were also tested by using the same method, and the concentration sensitivity reached −0.678 dB/(g/L) and −0.577 dB/(g/L), respectively, with the linear fit curves showing a high R^2^ of 97.8% and 98.2%, respectively, as shown in Figure 4f. The refractive index can be calculated as follows [32,33]:

(7) $$n_{g}=0.002015\times C_{g}+1.3292$$

*C_g_*: the concentration of the glucose solution and *n_g_* is the refractive index.

The refractive index sensitivities of the three probes were −267.49 dB/RIU, −336.48 dB/RIU and −286.35 dB/RIU, which were calculated according to Equation (7). *C_g_* was controlled by adjusting the injection time and flow rate of the dual-channel syringe pump. It is worth noting that the intensity of the interference dips of the HCSP also changed with the temperature. When the ambient temperature changed in the range from 32 °C to 37 °C, the intensity of the interference dip changed by 0.06 dB, as shown in Figure 4a. The temperature dependence of the interference dip loss was only 0.012 dB/°C due to the tiny CTE of SU-8. Therefore, the temperature crosstalk of the probe was so small that it could be neglected when measuring the solution concentration.

The center wavelength of the interference dip shifted by 0.06 nm when the concentration changed from 0.2 g/L to 1.2 g/L. The sensitivity caused by the change in RI was 0.06 nm/(g/L), as shown in Figure 4d, which was much less than the temperature sensitivity. The concentration cross can be neglected in the temperature measurement. Therefore, the detection of the liquid refractive index was realized by monitoring the change in the interference dip loss. Considering the spectrometer resolution was 0.02 nm and signal noise was 0.001 dB, the detection limit of the sensors can be calculated according to Ref. [34]. The detection limit of the temperature was 0.3 °C, and the detection limit of the concentration was 0.05 g/L.

## 4. Performance Test

**Figure 5:**
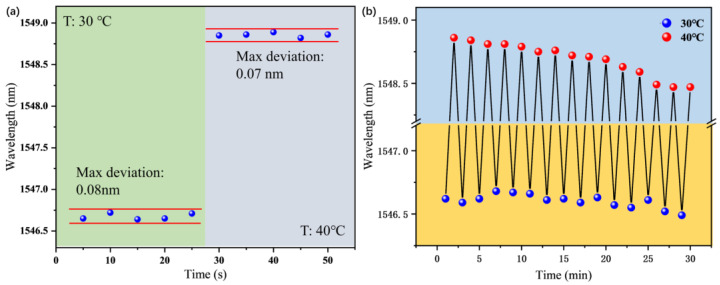
(**a**) Stability test of HCSP-1 between the temperature of 30 °C and 40 °C. (**b**) Repeatability test of HCSP-1 between the temperature of 30 °C and 40 °C.

The stability test was also performed. HCSP-1 was placed in a water bath at a temperature of 30 °C and 40 °C for 25 min. In Figure 5a, it can be seen that the maximum deviation of HCSP-1 was 0.08 nm, which included the reading error, the fitting error and the error caused by environmental changes. The repeatability test was also performed. HCSP-1 was regularly immersed in two water baths with temperatures of 30 °C and 40 °C. The shift of the wavelength dips was recorded in Figure 5b. Overall, the interference dip was stable except for a small jitter. It is worth noting that the interference dip shifted significantly toward the short wavelengths within 25 min, which was caused by the decrease in the ambient temperature. The probe showed good stability and repeatability.

**Figure 6:**
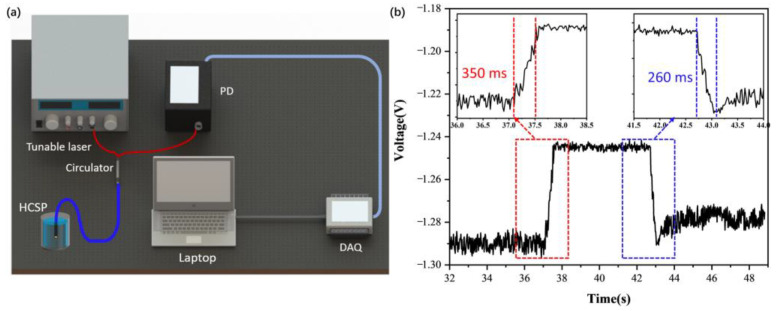
(**a**) The image of response time experimental setup. (**b**) The response time and recovery time of HCSP.

**Figure 7:**
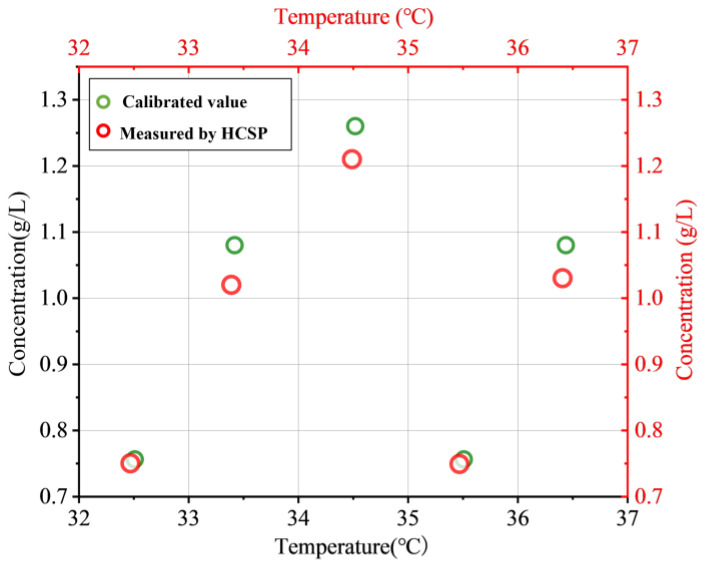
The difference between the calibrated value and value measured by the HCSP.

A tunable laser (OPEAK. Tech, TLS-3000, Shenzhen, China), a broadband source, a photoelectric detector (Thorlabs, DET08CFC, Shanghai, China) and a high-speed acquisition card (Smacq, USB-1000, Beijing, China) were used to study the response of the HCSP, as shown in Figure 6a. The response time was continuously recorded and the data were transferred to computers. The voltage fluctuated between the initial value and the high level (around the average value of the voltage) as the temperature changed. The rise time and recovery time of the HCSP were obtained. The rise time and recovery time of the flowmeter were 350 ms and 260 ms, respectively, as shown in Figure 6b. This shows that the HCSP responded quickly and could be used for real-time temperature monitoring. Finally, the biomimetic plasma was prepared with different concentrations of glucose. The concentration of the solutions was calibrated using a high-precision electronic balance and an Abbe index meter. Five groups of bionic blood samples with glucose concentrations of 0.756 g/L, 1.08 g/L, 1.26 g/L, 0.756 g/L and 1.08 g/L were set at 32.51 °C, 33.42 °C, 34.52 °C, 35.51 °C and 36.44 °C, respectively. The temperature of the biomimetic plasma solutions was calibrated with a commercially available electronic thermocouple with a resolution of 0.01 °C. HCSP-1 was used to monitor the glucose concentration and temperature of the plasma, but a small error was still present as shown in Figure 7. The results of five experiments and the calibrated values were averaged. It is easy to see that the maximum concentration difference was only 0.06 g/L and the temperature difference was only 0.04 °C. We believe that the difference was mainly due to the error of the solution ratio and curve fitting. The HCSP undoubtedly has great research value in the field of pathological monitoring, biopharmacy and bionic chip development.

The key parameters of the sensor probe were compared with other glucose fiber sensors reported in recent years, as shown in Table 1. The sensor probe proposed in this paper showed an excellent sensitivity and detection limit compared to other sensors. Additionally, the reflection-type structure has the advantages of small size and strong unity, which can be embedded in the microchips easily. It is suitable for the monitoring of the temperature and refractive index of microfluidics.

## 5. Conclusions

In this work, a microfluidic chip with integrated dual-parameter fiber-tip sensors is proposed. The effect of different probe shapes on the fluid field in microchannels was investigated. The HCSP was perfectly suited for the microfluidic chip. Multiple HCSPs were distributed in each channel of the chip, allowing for the real-time monitoring of the concentration and temperature characteristics of the microfluidic. The temperature sensitivity and concentration sensitivity could reach 314 pm/°C and −0.678 dB/(g/L), respectively. The temperature response of the spurious dip losses was only 0.012 dB/°C. The HCSPs were embedded in the channels using the high-precision five-dimensional displacement platform. The gaps between the fibers and the grooves were sealed with 535 thermosetting adhesives that were insensitive to temperatures. The integrated technology combines the optical fiber sensor with the microfluidic chip and is low cost with high performance. Therefore, we believe that the proposed microfluidic chip integrated with the optical sensing device is beneficial for drug discovery, pathological research and material science investigations. The technology integrated with the device has great application potential in μ-TAS.

## Figures and Tables

**Table 1:** Performance comparison of optic fiber glucose sensors reported in recent years.

Sensor Principle	Core Structure	Detection Limit (M)	Range (mM)	Sensitivity	Type	Reference
Mode filter	LFBG	0.139	0–10	0.64 dB/(g/L)	Transmission	[35]
	FBG	1 × 10^−9^	0–10	−	Reflection	[36]
	TFG	0.02	0–150	1.33 nm/(g/L)	Transmission	[37]
SPR	D-type PCF	–	0–100	0.83 nm/(g/L)	Transmission	[38]
	MMF	7.89 × 10^−4^	0–80	14 nm/(g/L)	Transmission	[39]
Mode interference	MMF+SMF	0.189	0–450	0.0267 nm/(g/L)	Transmission	[40]
	U-shape Fiber	0.011	1–5	3.123 nm/(g/L)	Transmission	[41]
	Single Cone SMF	0.278	0–300	1.74 nm/(g/L)	Reflection	[42]
	Polymer-cavity FP	2.8 × 10^−4^	0–6.6	−0.678 dB/(g/L)	Reflection	This work

## Data Availability

Not applicable.
